# Prevalence and Predictors of Stunting among Primary School Children in Northeast Ethiopia

**DOI:** 10.1155/2021/8876851

**Published:** 2021-06-25

**Authors:** Getaw Walle Bazie, Mohammed Seid, Gudina Egata

**Affiliations:** ^1^Department of Epidemiology and Biostatistics, School of Public Health, College of Medicine and Health Sciences, Wollo University, Dessie, Ethiopia; ^2^School of Public Health, College of Health Sciences, Addis Ababa University, Addis Ababa, Ethiopia

## Abstract

**Background:**

Stunting is a major public health problem affecting children in low- and middle-income countries and has become one of the underlying causes of early childhood mortality. However, there is a paucity of information on the prevalence of stunting and its predictors among school children in these settings including Ethiopia.

**Objective:**

The aim of this study was to assess the prevalence of stunting and its predictors among school children in Northeast Ethiopia.

**Methods:**

A school-based cross-sectional study design was used among 341 primary school children in Northeast Ethiopia from October to December 2019. A simple random sampling technique was used to recruit the study subjects. A pretested structured questionnaire was used to collect sociodemographic and dietary data. Anthropometric data were generated using WHO AnthroPlus software version 1.0.4. Binary logistic regression analysis was used to see the association between independent variables and the outcome variable. Odds ratio along with 95% confidence interval was estimated to measure the strength of the association. The level of statistical significance was declared at *p* value ≤ 0.05.

**Results:**

The prevalence of stunting was found to be 14.1% (95% CI: 10.1%, 18.1%). Family size of 6–9 (AOR = 2.43; 95% CI: (1.16, 4.58)), washing hands less frequently before eating (AOR = 3.96; 95% CI: (2.09, 11.66)), and intestinal parasitic infection (AOR = 2.66; 95% CI: (1.16, 4.95)) were significantly associated with stunting.

**Conclusion:**

The prevalence of stunting among school-age children was a great public health concern. Large family size, poor handwashing practice before meals, and intestinal parasitosis were significant predictors of stunting. Thus, periodic deworming, health education on personal hygiene, and health promotion and counseling on family planning need to be strengthened by all relevant stakeholders.

## 1. Introduction

Stunting is defined as a measure of linear growth retardation in which a child is short for his or her age below −2 standard deviations (SD) of the height-for-age median value of the world health organization (WHO) child growth standards [1, 2]. It is a result of chronic malnutrition and is one of the public health problems worldwide. Undernutrition alone is responsible for more than one-third of child deaths globally and accounts for eleven percent of the global burden of disease. It is more prevalent in low- and lower-middle-income countries. Child undernutrition is a collective adverse consequence which is recognized later in life, and it includes impaired cognitive development, poorer educational achievement, and human capital formation. It is associated with poor developmental achievement in young children and poor school performance in older children [3, 4].

Stunting among school-age children is becoming one of the major public health concerns worldwide. Out of 667 million children aged under 5, 159 million are too short (stunted) for their age [5] and 52% of them are living in developing countries [3]. About 46.5% of these children are from sub-Saharan Africa of which half of them are severely stunted [4].

Stunting is widely assumed to occur mainly in early childhood by the age of three years. Children stunted at school age are likely to have a history of poor nutritional status. However, children can exhibit catch-up growth if their environment improves which suggests that interventions among school-age children can supplement efforts in the preschool years to reduce levels of stunting and related effects on children's health and education [6].

Stunting rates among young children are the highest in sub-Saharan Africa [7, 8]. As school age is a period of physical and mental development, prolonged undernutrition in this age group impairs their growth. It could also increase the susceptibility of children to disease. Despite continued prevention efforts, child undernutrition remains a major public health problem in sub-Saharan Africa, including Ethiopia [9, 10].

In Ethiopia, stunting is one of the most serious public health problems [11]. High stunting rates in the country pose a significant obstacle to achieving better child health outcomes. According to the Ethiopian Demographic Health Survey, stunting is greater among children in rural areas (40%) than urban areas (25%) in Ethiopia [12].

Stunting is associated with an increased risk of mortality during childhood [13, 14]. Even when it does not cause death, stunting can impose lifelong damage on a child's health and development [15]. The negative consequences also have great effects on the economies where s/he lives, learns, and works. Other consequences include the following: during schooling years, stunted children are more likely to repeat grades and drop out of school, reducing, thus, their income-earning capability later in life. Furthermore, adults who were stunted as children are less likely to achieve their expected physical and cognitive development, thereby impacting their productivity. Although Ethiopia has made important progress in reducing the current levels of stunting in children, a large proportion of the adult population is currently living with the lifelong consequences of childhood stunting rates that reached more than half of the population [16].

Good nutrition is vital for cognitive and physical development having both short- and long-term impacts on the quality of life [17]. Children must be healthy and well-nourished in order to fully participate in education and gain its maximum benefits. Early childhood care programs and primary schools that advance children's health and nutrition can enhance the learning and educational outcomes of school children [18]. Thus, improving the nutrition of women and children would help in overcoming some of the greatest health challenges facing the world in many ways such as the burden of chronic and degenerative diseases and maternal mortality among others.

Although the problem of malnutrition in Ethiopia is somewhat well documented, regional or zonal specific data particularly with regard to the relative contribution of different predictors to stunting among school children are lacking to design appropriate interventions. In addition, as one of the historically drought-affected areas in Ethiopia to break down the problems associated with stunting and establish effective nutrition services for school children, which is the key element in the prevention of undernutrition, up-to-date information is needed. Up to the present time no single study has been conducted in the study area which assessed the nutritional status as stunting and its predictors. Therefore, this study was aimed at assessing the prevalence of stunting and its predictors among school-age children in the study setting.

## 2. Materials and Methods

### 2.1. Study Design and Area

A school-based cross-sectional study design was used among school children in Kutaber district, Northeast Ethiopia, from October to December 2019. Kutaber district has a 95,410 population of which 90,470 (94.8%) live in a rural setting and 4,940 (5.2%) are urban dwellers. The livelihood of the surrounding population is heavily dependent on crop and livestock production. The major food crops are cereals and grains grown in temperate-highland areas. Sweet potato and onion are the major cash crops grown in the area [19]. There are 2 governmental primary schools in the district with a total student population of 2,108.

### 2.2. Study Population

The study population was all primary school children who were available during data collection in selected primary schools. All children who were enrolled in the primary schools in Kutaber district and living at least for 6 months prior to the study in the district were included in the study. Children with physical deformity who might distort the quality of anthropometric measurement and those who had taken deworming treatment one month prior to the survey were excluded from the study.

### 2.3. Sample Size and Sampling Procedures

The sample size was computed using family size as a factor [20] associated with stunting using Epi-Info™ 7 with the following assumptions: Confidence level of 95%, power of 80%, odds ratio of two, proportion of stunting among high family size group 46%, and 10% nonresponse. The study was conducted in the 2 governmental primary schools (30% of all the primary schools) found in Kutaber district. The total number of students in the two primary schools was 2,108 and the final sample size was 341. The samples were proportionally allocated to each grade to their size in both schools. Finally, students from each grade level were recruited by simple random sampling technique using the school roster as the sampling frame.

### 2.4. Study Variables

The dependent variable was stunting (height-for-age <−2SD). The independent variables were sociodemographic variables (age, etc.), environmental variables (water source, etc.), intestinal parasites (*Entamoeba hystolitica*, etc.); disease (infection) (pneumonia, etc.), and dietary intake.

### 2.5. Operational Definitions

  Stunting: defined as a child whose height-for-age z-score (HAZ) was below −2SD units using new WHO child growth standards [1]. All stunted children were coded as “1” and “0” otherwise for analysis.
  Dietary diversity score (DDS): dietary intake and dietary diversity scores of the school-age children were examined through 24 hours dietary recall of the foods commonly consumed by the study participants. There were 17 food items grouped into eight as grains/roots or tubers, vitamin A-rich plant foods, other fruit or vegetables, meat/poultry/fish-seafood, eggs, pulses/legumes/nuts, milk and milk products, and food cooked in oil/fat. DDS was collected and calculated as the sum of the number of different food groups consumed by the respondent in the 24 hours prior to the assessment. The dietary diversity score (DDS) was calculated by giving a score of “1” for those who consumed the food item and a score of “0” for those who did not consume the food item over the past 24 hours prior to the study. Then, DDS was ranked and divided into three subgroups as low, moderate, and excellent.
  Low: if they ate three or fewer items of the above eight food item groups.
  Moderate: if they ate four to five items of the above eight food item groups.
  Excellent: if they ate six to eight items of the above eight food item groups.

### 2.6. Data Collection Procedures and Quality Control

The questionnaire was initially prepared in English and then translated into the local language, “Amharic,” and retranslated back to English by fluent speakers of both languages to ascertain its consistency. Data were collected by five bachelor holder nurses who had similar exposure in data collection in the past and the process of data collection was supervised by two supervisors. Sociodemographic data were collected using a pretested and structured interviewer-administered questionnaire while children's height was measured using Seca Germany model 755, Ser. No. 5755322115250 height scale and each child's age was taken from the school's roster [21]. A 24-hour dietary recall questionnaire was used to assess the children's dietary intake in the previous 24 hours. Dietary intake and dietary diversity scores of the school-age children were examined through 24-hour dietary recall of the foods commonly consumed by the study participants. There were 17 food items grouped into eight as grains/roots or tubers, vitamin A-rich plant foods, other fruit or vegetables, meat/poultry/fish/seafood, eggs, pulses/legumes/nuts, milk and milk products, and food cooked in oil/fat.

Fecal samples were collected by two laboratory technicians using sterile, dry, well-labeled disposable plastic stool cups by their codes and spoon distributed to each study participant along with brief instructions on how to collect the stool. Students were advised to fill up the disposable plastic cup about the size of the tip of the thumb (approximately 5 grams of fresh stool) using a disposable spoon that was given with the container. The stool sample was preserved using 10% formaldehyde concentration and transported to Dessie Referral Hospital for parasitological examination and enumeration. The direct wet mount technique was used by three laboratory technologists to assess the overall prevalence of intestinal parasitic infections in the study area. The direct wet mount was processed by conventional normal saline to identify the presence of motile intestinal parasites, cysts, egg, and trophozoite under an electron microscope at 10x and 40x magnification. Saline was used to observe cysts of intestinal parasites. About 2 g of stool samples were emulsified with 3-4 ml normal saline, then a drop of the emulsified sample was placed on a clean microscopic glass slide, and a few drops of normal saline solution were added and covered with a coverslip. Finally, the results were recorded in the appropriate form by their code and were analyzed.

To keep the quality of data, microscopic reading was examined by senior laboratory technologists. Data quality was also assured by prior training of data collectors and supervisors about the objectives of the study and data collection procedures. Anthropometric data were taken by digital SECA Germany measuring tools, and each child was checked without shoes, with the least possible clothes, with empty pockets, and with no earrings and measured two times. The average weight or height was taken and strict supervision was ensured while taking the measurements.

The questionnaire was well designed and formatted ahead of data collection. A pretest was done on 5% of the estimated sample size outside the study locality to see the consistency and accuracy of the study tools. Height measurement was standardized using reference anthropometric measurer before deploying the data collectors to the field to minimize the technical error of measurement (TEM).

### 2.7. Data Processing and Analysis

The collected data were entered into Epi-Data version 3.02 software and exported to SPSS version 20 for analysis. The SD (*z*-score) for anthropometric data was generated using WHO AnthroPlus software version 1.0.4. Descriptive statistics were used to summarize the data. Bivariable logistic regression analyses were used to track the association between each independent variable and stunting. Variables with a *p* value less than 0.25 during bivariable analyses were retained and entered into the final multiple logistic regression model to control for all possible confounders. The goodness-of-fit test was checked using the Hosmer–Lemeshow statistical significance test while multicollinearity between independent variables was checked by determining variance inflation factor (VIF) [14]. The values for both tests were greater than 0.05 and less than 10%, respectively. Odds ratio along with 95% CI were estimated to measure the strength of the association between variables. The level of statistical significance was declared at *p* value  ≤  0.05.

### 2.8. Ethical Considerations

The study was conducted following the Declaration of Helsinki. Ethical clearance was obtained from the Institutional Review Board of the College of Medicine and Health Sciences, Wollo University. A permission letter to conduct the study was obtained from the Kutaber district education office. The objective of the study was explained to the teachers and students at the time of specimen collection. Written informed consent was obtained from every selected participant's parent or guardian and assent was secured from the children themselves before conducting the survey. Any involvement in the study was done after their complete consent was obtained. They were informed that all the data obtained from them would be kept confidential by using codes instead of any personal identifiers and was meant only for the study. Their rights to refuse participation any time they want were assured. The students found to be infected with intestinal parasites during data collection were referred to nearby health facilities to be diagnosed.

## 3. Results

### 3.1. Sociodemographic Characteristics of School-Age Children

A total of 341 primary school children participated in the study. Nearly 53% of them were males. The mean (±SD) age was 11.4 (±2.54) years. About 263 (77.1%) of the respondents were Muslims. More than half (58.4%) of the students belonged to grades 5–8. Nearly 54% of the students were from households with large (6–9) family sizes (Table 1).

### 3.2. Environmental Characteristics of School-Age Children

According to the school-age children report, the main source of drinking water was piped water (68.3%) followed by protected well (22.9%). About 96.5% had latrine for the household and have been using latrine to defecate. With regard to solid waste disposal, 233 (68.3%) disposed of their household waste in the pit followed by 63 (18.5%) disposed of their household waste by open dumping. With regard to handwashing, 11.4% of school-age children had no handwashing practice before eating. With regard to materials used for handwashing, 284 (83.3%) used water and soap followed by 54 (15.8%) who used only water. Regarding dirty matter in their fingers, 210 (61.6%) had dirty matters in their fingers (Table 2).

### 3.3. Prevalence of Intestinal Parasitic Infections

The overall prevalence of intestinal parasitic infections among primary school children was 24.3%, 95% CI: (19.9%, 29.0%). The predominant species of parasites among school children were *E. hystolitica* (71.10%) and *G. lamblia* (25.3%) (Table 3).

### 3.4. Prevalence of Stunting among Primary School Children

Anthropometric measurements of children were converted to height-for-age *z*-score. The mean (±SD) *z*-score was −0.88 (±1.1). The overall prevalence of stunting was 14.1%, 95% CI: (10.1%, 18.5%) of which 2.64% of the students were severely stunted. Boys were found to be more stunted (56.3%) compared with females (43.8%) (Table 4).

### 3.5. Predictors of Stunting among Primary School Children

In the bivariable logistic regression, age, family size, handwashing practice before meals, handwashing practice after defecation, and intestinal parasitic infection were found to be significant with *P* value less than or equal to 0.25 and entered to multivariable logistic regression. Finally, in the multivariable logistic regression analysis, family size of 6–9 (AOR = 2.43; 95% CI: (1.23, 4.88)), less frequent handwashing practice before eating meal (AOR = 3.96; 95% CI: (1.745, 8.97)), and being infected with parasites (AOR = 2.66; 95% CI: (1.30, 5.41)) were significantly associated with stunting after adjusting for all possible confounders.

The odds of stunting were 2.42 times higher among children from households having a large family size compared with those children from households with less family size. The odds of stunting were 3.96 times higher among children from families who less frequently practice handwashing before eating meals compared with their counterparts. Moreover, the odds of stunting were nearly 2.7 times higher among children who have been infected with parasites compared with noninfected ones (Table 5).

## 4. Discussion

The study aimed to assess the prevalence of stunting and its predictors among school-age children in Kutaber district, Northeast Ethiopia. The prevalence of stunting was found to be 14.1% in this study. Large family sizes, handwashing practice before eating, and being infected with intestinal parasites were significant predictors of stunting.

The prevalence of stunting observed in this study is in line with the result of one previous study in Northwest Ethiopia [20]. However, it is lower than the results of numerous previous studies done across the world including Ethiopia ranging from 27% to 60% [22–25] but higher than the study done in Pune, India [26]. These differences might be due to variations in sample size, socioeconomic differences, cultural and dietary practice, and other geographic-related factors.

Children who were born to households with large family sizes were more likely to be stunted compared with their counterparts. This finding was similar to the study conducted among similar populations in Northwest Ethiopia [22, 23]. This might be because, in large size family, children might not get enough food. In addition, the more the family size, the less likely children eat a variety of food items. This in turn could have put children at higher risk of stunting. This finding implies balancing family size to effectively utilize resources and protect children to be healthy is very important.

Children who did not practice handwashing before meals were more likely to be stunted compared with their counterparts. This finding was in accordance with a previous study conducted in Ethiopia among a similar study population in Ethiopia [24]. This might be attributed to improper personal hygiene that might expose the children to intestinal parasitic and other recurrent infections that could have resulted in malnutrition due to malnutrition infection vicious cycle.

In this study, it was documented that there is a significant association between intestinal parasitic infections and stunting. Children who were infected with parasites were more likely to be stunted compared with their noninfected counterparts. This finding was in agreement with the results of other studies conducted in Africa including Ethiopia [25, 27]. This might be because intestinal parasitic infection competes for human nutrient uptake and impairs the immune system of the host so that it makes them susceptible to many infections.

Overall, the findings of this study imply that limiting family size, frequently washing hands before meals, and protecting children from conditions that might expose them to different parasites is very important to improve the nutritional status of children.

Taking a single 24-hour recall dietary data is one of the limitations of the current study since it might not reflect the usual intake and all-important diet-related predictors were not assessed. We recommend other researchers to apply the multiple pass 24-hour recall method. Moreover, involving the parents to respond to some of the questions would have been advantageous but it was not feasible during data collection. The cross-sectional nature of the study is the other limitation that did not enable us to establish strong causal relationships between the predictors and stunting. Therefore, we recommend other investigators to apply other study designs that include control groups such as cohort study that could establish strong causal relationships between the predictors and stunting.

The findings of this study could be generalized to primary school children attending at rural districts of Ethiopia since more or less the feeding practice of primary school children is similar.

## 5. Conclusions

The prevalence of stunting among school-age children was a great public health concern (nearly one out of eight school-age children are stunted) that needs the intervention of different stakeholders to secure the nutritional status of school-age children. Large family size, poor handwashing practice before meals, and intestinal parasitosis were significant predictors of stunting. Thus, it is to be noted that handwashing practice, water, sanitation and hygiene (WASH), school feeding, and deworming programs should be integrated and implemented in school health services so as to bring behavioral change and promote the health of school children by overcoming the effects of malnutrition. Moreover, relevant stakeholders should also pay due attention to the incorporation periodic deworming program in a national nutrition program to achieve the Sustainable Development Goals and health education on personal hygiene and health promotion and counseling on family planning need to be strengthened.

## Figures and Tables

**Table 1:** Sociodemographic characteristics of primary school children in Kutaber district, Northeast Ethiopia, 2019 (*n* = 341).

Characteristics	Categories	Number (%)
Age (years)	6–9	82 (24.05)
	10–14	230 (67.45)
	15–17	29 (8.5)

Sex	Male	177 (51.9)
	Female	164 (48.1)

Residence	Rural	165 (48.4)
	Urban	176 (51.6)

Religion	Muslim	263 (77.1)
	Orthodox	78 (22.9)

Ethnicity	Amhara	339 (99.4)
	Others^*∗*^	2 (0.6)

Grade level	1–4	142 (41.6)
	5–8	199 (58.4)

Mother's education	Have no formal education	100 (29.3)
	Read and write	31 (9.1)
	Elementary	104 (30.5)
	Secondary and above	106 (31.1)

Parent's condition	Mother dead	5 (1.5)
	Father dead	13 (3.8)
	Both dead	3 (.9)
	Both alive and live together	292 (85.9)
	Live separately	19 (5.6)
	Divorced	9 (2.6)

Living condition of the child	Live with family	315 (92.4)
	Live outside family	26 (7.6)

Mother's occupation	Housewife	222 (65.1)
	Merchant	49 (14.4)
	Government employee	49 (14.4)
	Private employed	18 (5.3)
	Others^*∗∗*^	3 (0.9)

Father's education	Have no formal education	52 (15.2)
	Read and write	38 (11.1)
	Elementary	104 (30.5)
	Secondary and above	134 (39.3)

Father's occupation	Farmer	137 (40.2)
	Merchant	64 (18.8)
	Government employee	86 (25.2)
	Private employee	33 (9.7)
	Others	9 (2.3)

Family size	1–5	226 (66.3)
	6–9	115 (33.7)

Others ^*∗*^ = Tigre and Oromo; others ^*∗∗*^ = daily laborer and farmer.

**Table 2:** Environmental characteristics of primary school children in Kutaber district, Northeast Ethiopia, 2019 (*n* = 341).

Characteristics	Categories	Frequency (%)
Source of drinking water	Pipe water	233 (68.3)
	Protected spring	9 (2.6)
	Unprotected spring	4 (1.2)
	Protected well	78 (22.9)
	Unprotected well	17 (5)

Place of defecation	Latrine	329 (96.5)
	Open	8 (2.3)
	River	4 (1.2)

Solid waste disposal	Open dumping	63 (18.5)
	Burning	45 (13.2)
	Pit	233 (68.3)

Handwashing practice before eating	Frequent	302 (88.6)
	Infrequent	39 (11.4)

Handwashing after defecation	Frequent	240 (70.4)
	Infrequent	101 (29.6)

Materials used for handwashing	Water only	54 (15.8)
	Water and soap	284 (83.3)
	Water and ash	3 (0.9)

Dirty matter on fingers	Absent	131 (38.4)
	Present	210 (61.6)

Shoe wearing practice	Always	334 (97.9)
	Sometimes	7 (2.1)

**Table 3:** Prevalence of intestinal parasitic infections among school-age children in Kutaber district, Northeast Ethiopia, 2019 (*n* = 83).

Parasites	Number	Percent (%)
*E. hystolitica*	59	71.10
*G. lamblia*	21	25.30
*A. lumbricoides*	2	2.41
*H. nana*	1	1.20
Total	83	100

**Table 4:** Prevalence of stunting by gender among primary school children in Kutaber district, Northeast Ethiopia, 2019 (*n* = 341).

Nutritional status	Height-for-age by gender					
	Girls		Boys		Total	
	Frequency	%	Frequency	%	Frequency	%
<−2SD	21	43.75	27	56.25	48	100
−2SD to 2SD	138	48.25	148	52.67	286	100
>2SD	3	42.86	4	57.14	7	100
Total	162	47.51	179	52.49	341	

**Table 5:** Predictors of stunting among primary school children in Kutaber district, Northeast Ethiopia, 2019.

Variables	Category	Stunting		COR (95% CI)	AOR (95% CI)
		Yes	No		
Age (years)	6–11	10	118	0.63 [0.34, 1.159]	0.52 [0.26, 1.02]
	12–17	38	115	1	1

Family size	1–5	22	204	1	1
	6–9	26	89	2.71 [1.457, 5.04]	2.43 [1.228, 4.79]^*∗*^

Handwashing practice before eating	Infrequent	17	22	6.76 [3.24, 14.07]	3.96 [1.75, 8.97]^*∗*^
	Always	31	271	1	1

Handwashing practice after defecation	Sometimes	24	77	2.81 [1.505, 5.23]	2.17 [0.070, 4.42]
	Always	24	216	1	1

Parasitic infection	Yes	22	61	3.22 [1.707, 6.07]	2.66 [1.304, 5.41]^*∗∗*^
	No	26	232	1	1

^*∗*^ *p* value <0.05. ^*∗∗*^*p* value <0.01.

## Data Availability

The data supporting the findings of this study are available from the corresponding author upon request.
